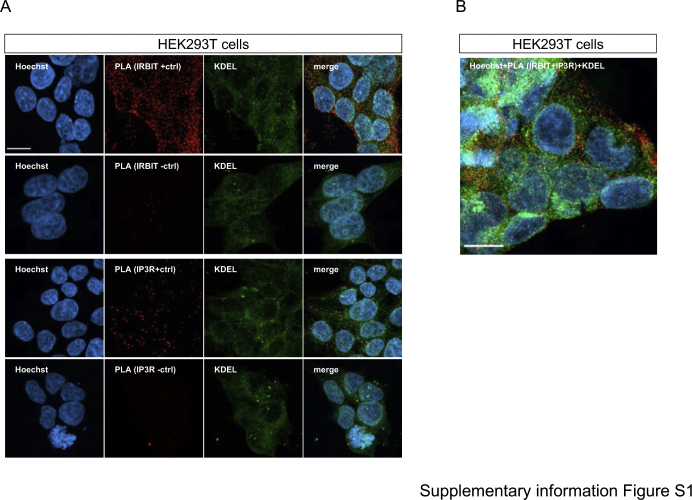# Unbiased proteomic profiling reveals the IP3R modulator AHCYL1/IRBIT as a novel interactor of microtubule-associated protein tau

**DOI:** 10.1016/j.jbc.2022.101774

**Published:** 2022-02-24

**Authors:** Lena Wischhof, Aasha Adhikari, Mrityunjoy Mondal, Anaïs Marsal-Cots, Jacek Biernat, Eva Maria Mandelkow, Eckhard Mandelkow, Dan Ehninger, Pierluigi Nicotera, Daniele Bano

**Affiliations:** 1 German Center for Neurodegenerative Diseases (DZNE), Bonn, Germany; 2 CAESAR Research Center, Bonn, Germany; 3 Department of Neurodegenerative Diseases and Geriatric Psychiatry, University of Bonn, Bonn, Germany

**Keywords:** Alzheimer’s disease, autophagy, human protein microarray, microtubule-associated protein tau, SAH hydrolase–like protein 1 (AHCYL1/IRBIT), AD, Alzheimer's disease, AHCY, SAH hydrolase, AHCYL1/IRBIT, SAH hydrolase–like protein 1/IP3R-binding protein, co-IP, coimmunoprecipitation, DMEM, Dulbecco's modified Eagle's medium, ER, endoplasmic reticulum, HA, hemagglutinin, HEK293T, human embryonic kidney 293T cell line, IgG, immunoglobulin G, IP3R, inositol 1,4,5-trisphosphate receptor, iPSC, induced pluripotent stem cell, MAP, microtubule-associated protein, OCR, oxygen consumption rate, O/E, overexpression, Pen/Strep, penicillin/streptomycin, PLA, proximity ligation assay, RIPA, radioimmunoprecipitation assay, smNPC, small-molecule neural progenitor cell, TBS, Tris-buffered saline

## Abstract

Microtubule-associated protein tau is a naturally unfolded protein that can modulate a vast array of physiological processes through direct or indirect binding with molecular partners. Aberrant tau homeostasis has been implicated in the pathogenesis of several neurodegenerative disorders, including Alzheimer’s disease. In this study, we performed an unbiased high-content protein profiling assay by incubating recombinant human tau on microarrays containing thousands of human polypeptides. Among the putative tau-binding partners, we identify SAH hydrolase–like protein 1/inositol 1,4,5-trisphosphate receptor (IP3R)–binding protein (AHCYL1/IRBIT), a member of the SAH hydrolase family and a previously described modulator of IP3R activity. Using coimmunoprecipitation assays, we show that endogenous as well as overexpressed tau can physically interact with AHCYL1/IRBIT in brain tissues and cultured cells. Proximity ligation assay experiments demonstrate that tau overexpression may modify the close localization of AHCYL1/IRBIT to IP3R at the endoplasmic reticulum. Together, our experimental evidence indicates that tau interacts with AHCYL1/IRBIT and potentially modulates AHCYL1/IRBIT function.

Microtubule-associated protein (MAP) tau is a highly soluble and natively unfolded molecule that normally binds microtubules and contributes to regulation of axonal transport in neurons (1, 2, 3). Tau has four structurally and functionally defined domains that determine its intrinsic promiscuous target behavior. The central core of the protein (residues ∼150–370) consists of a basic microtubule-binding domain containing a proline-rich region with recognition sites for molecules critically involved in cell signaling, plus three or four imperfect repeat sequences involved in attachment to microtubules and formation of pathological paired helical filaments in Alzheimer’s disease (AD) (4, 5, 6). The acidic N-terminal region (residues ∼1–150) projects into the cytosol and interacts with various cofactors, for example, motor proteins or the plasma membrane through annexin A2 (7, 8), whereas the C-terminal tail region (residues ∼370–441) regulates microtubule assembly and remodeling (4, 9, 10, 11). In the human adult central nervous system, alternative splicing of the same pre-RNA results in several isoforms that migrate on SDS-PAGE with an apparent molecular weight between 60 and 72 kDa (12). These six isoforms are differently expressed during neurogenesis and neural differentiation, with the longest splicing variant (2N4R, hereafter referred to as htau40) as the most abundant tau isoform in axons of fully differentiated neurons (12, 13, 14, 15). A large body of evidence has demonstrated a correlation between aberrant tau biology (*e.g.*, expression, intracellular localization, and formation of inclusions, oligomers, and aggregates) and a heterogeneous group of age-related neurodegenerative diseases generally referred to as tauopathies (1, 2, 3, 16, 17). A common hallmark of tauopathies is the presence of intracellular insoluble deposits of abnormally modified tau, which tends to form paired helical filaments (18) as neurofibrillary tangles (19, 20) in the neuronal soma and dendrites. While inherited cases of frontotemporal dementia with parkinsonism linked to chromosome 17 emphasize the importance of pathological tau as one of the main causes of “primary tauopathies” (21, 22, 23), the association of aberrantly modified tau with the clinical and neuropathologic etiology of “secondary tauopathies” (*e.g.*, Niemann–Pick disease type C, traumatic brain injury, and chronic traumatic encephalopathy) is more loose and mainly correlated to the presence of neuronal and/or astrocytic tau aggregates in certain brain areas (2, 17). Based on familial cases of frontotemporal dementia with parkinsonism linked to chromosome 17, Pick's diseases, corticobasal degeneration, and progressive supranuclear palsy (24, 25), disease-causing mutations in the *MAPT* (MAP tau) gene lead to heterogeneous molecular consequences. In this regard, most tau variants exhibit a higher tendency to form insoluble filaments and have different affinities for interacting partners or subcellular structures (*e.g.*, microtubule, plasma membrane). Most relevant histopathological and clinical features include the pronounced presence of post-translationally modified tau in various brain areas, cognitive impairments, diffuse atrophy of the neocortex, and medial temporal lobe associated with neurodegenerative processes (17). In patients affected by sporadic forms of AD, tau is detectable at early stages in neuronal dendrites and soma of neuroanatomically connected brain areas, such as the entorhinal cortex and the hippocampus (26, 27). In this regard, aberrant tau phosphorylation may lead to its missorting in the dendritic compartment, impairing the maintenance of dendritic structures and, consequently, undermining postsynaptic structures (28, 29, 30). Since dendritic spine remodeling correlates with the cognitive performance of an animal (31, 32), it is reasonable to believe that tau-mediated dysregulation of these subcellular structures may contribute to the cognitive decline of patients with tauopathies. As such, the pathogenic contribution of aberrant tau biology in AD remains a long-lasting research priority (1, 3, 16, 33).

While a large number of studies have focused on tau in many pathological settings, less is known about the physiological function of tau beyond its role in microtubule stabilization and axonal transport (3). In this regard, because of its intrinsic properties and binding affinity to a large spectrum of proteins (34), tau may hijack regulators of metabolic pathways and signaling cascades that orchestrate critical biological processes, as previously described for phosphatase and tensin homolog and the downstream insulin pathway (35). Therefore, it seems plausible that physiological as well as pathological mislocalization of tau may shift tau promiscuous binding properties toward unconventional partners, expanding the tau regulatory network. Consistently, the identification of novel interactors and modifiers of tau homeostasis may help to understand the complex biology of this unique MAP in cell physiology.

In an attempt to address this need, we carried out an unbiased *in vitro* high-content protein profiling of putative htau40 interactors. We identified SAH hydrolase–like protein 1/inositol 1,4,5-trisphosphate receptor (IP3R)–binding protein (AHCYL1/IRBIT) as a novel putative tau-interacting protein in cultured cells. Our study reveals a previously unknown tau interactor that might modulate intracellular processes, thereby broadening the pathophysiological importance of tau in various aspects of cell biology.

## Results

### AHCYL1/IRBIT is a novel tau-binding partner

**Figure 1:**
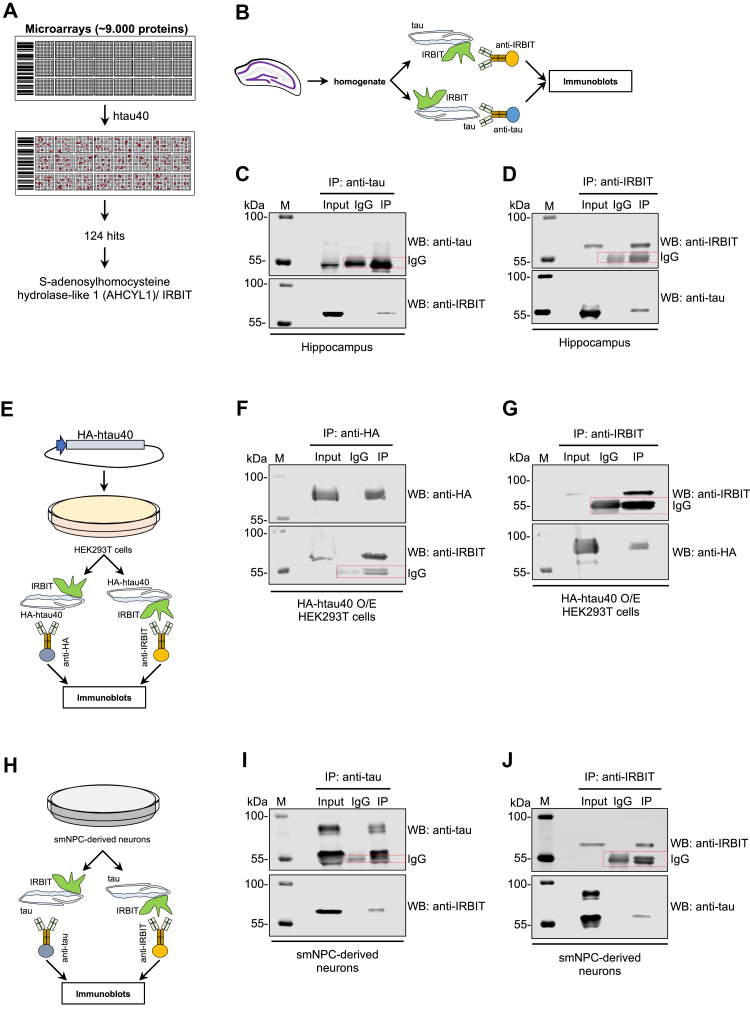
**AHCYL1/IRBIT binds tau.***A*, schematic representation of the human protein–protein microarray approach. Using 5 and 50 ng/μl of recombinant full-length human tau, we identified 124 tau-interacting polypeptides, including AHCYL1/IRBIT. *B*, scheme and (*C* and *D*) WBs of immunoprecipitated proteins from mouse adult hippocampi. Immunoblots were developed using anti-tau and anti-IRBIT antibodies. *E*, scheme and (*F* and *G*) WBs of immunoprecipitated proteins from HEK293T cells transiently transfected with a plasmid encoding HA-tagged htau40. After 48 to 72 h, cells were harvested, crosslinked, and lysed. Co-IPs were performed using an antibody against (*F*) HA and (*G*) IRBIT. *H*, scheme of IP strategy and (*I* and *J*) WBs of immunoprecipitated proteins from smNPC-derived neuronal cultures. Co-IPs were performed using antibodies against (*I*) tau and (*J*) IRBIT. Immunoblots were developed using the antibodies indicated on the *right*. *Red dotted rectangles* indicate bands because of IgG. M is protein molecular marker. AHCYL1/IRBIT, SAH hydrolase–like protein 1/IP3R-binding protein; co-IP, coimmunoprecipitation; HA, hemagglutinin; HEK293T, human embryonic kidney 293T cell line; IgG, immunoglobulin G; smNPC, small-molecule neural progenitor cell; WB, Western blot.

With the purpose of finding novel molecular interactors of tau, we employed high-content protein microarrays for screening of functional polypeptides that can physically bind recombinant htau40 (Fig. 1*A*). Using 50 and 5 ng of *Escherichia coli*–derived recombinant human full-length tau protein (36), we detected 124 polypeptides that interact with htau40 in our experimental conditions (Fig. 1 and Table S1). For 121 sequences, we could confirm the annotated ORF ID across multiple databases (Table S1). Among them, DNAJ/heat shock protein 40 kD, S100 calcium-binding protein B, and bridging integrator 1 were previously described as *bona fide* tau interactors (37, 38, 39, 40), highlighting the quality of our *in vitro* proteomic analysis. Two additional polypeptides, Src substrate cortactin and MAP RP/EB family member 2, were also retrieved in a recently reported tau interactome in induced pluripotent stem cell (iPSC)–derived glutamatergic neurons (41). We focused our study on AHCYL1/IRBIT, a protein that modulates IP3R activity (42, 43, 44) and therefore with a potential role in mitochondrial bioenergetics that may be relevant in tauopathies as recently emphasized (41). It is known that the C-terminal region of AHCYL1/IRBIT is highly homologous to the adenosylhomocysteinase AHCY, whereas the N-terminal region (residues 1–105) is unique for the IRBIT family members across phyla (44, 45). Since recombinant htau40 could bind two AHCYL1/IRBIT polypeptides in our microarrays (Table S1), we sought to confirm the physical interaction between tau and AHCYL1/IRBIT by using conventional coimmunoprecipitation (co-IP) assays. As a first step, we performed co-IPs using hippocampi from adult mice (Fig. 1*B*). Lysates were incubated with primary antibodies against tau or IRBIT, and immunoprecipitated proteins were immunoblotted to assess IRBIT and tau, respectively (Fig. 1, *C* and *D*). Consistent with a possible physical interaction as shown *in vitro* (Fig. 1*A*), we found that tau and IRBIT could immunoprecipitate each other from hippocampus lysates (Fig. 1, *C* and *D*). To substantiate this line of evidence, we overexpressed hemagglutinin (HA)-tagged htau40 in human embryonic kidney 293T (HEK293T) cells (Fig. 1*E*), which have been widely used in the field to assess tau post-translational modifications and interactions (46). After exposing cells to the reversible crosslinker dithiobis(succinimidyl propionate), we lysed the cells and performed co-IPs using a primary antibody against HA. Immunoblot analysis revealed a substantial amount of endogenous AHCYL1/IRBIT in the co-IP cell extracts (Fig. 1*F*). Reciprocal co-IP with AHCYL1/IRBIT antibody pulled down HA-tagged tau in cell homogenates (Fig. 1*G*). These data show that tau physically interacts with AHCYL1/IRBIT in transiently transfected HEK293T cells. To further support our findings in an additional cellular system, we employed human iPSC, from which we generated small-molecule neural progenitor cells (smNPCs) that were then spontaneously differentiated into neuronal cultures. Since these cells express high levels of tau isoforms normally associated with embryogenesis (13, 14, 15), we used an antibody (Tau46) recognizing the common C-terminal domain (residues 404–441) of tau, which enabled the efficient co-IP of endogenous AHCYL1/IRBIT (Fig. 1,*H* and *I*). Consistently, reciprocal co-IP using AHCYL1/IRBIT antibody could retrieve endogenous tau in homogenates from smNPC-derived neurons (Fig. 1*J*). Taken together, these biochemical analyses suggest that tau can physically bind AHCYL1/IRBIT in a protein microarray as well as in cultured cells.

### Tau is in close proximity to AHCYL1/IRBIT in cultured cells

**Figure 2:**
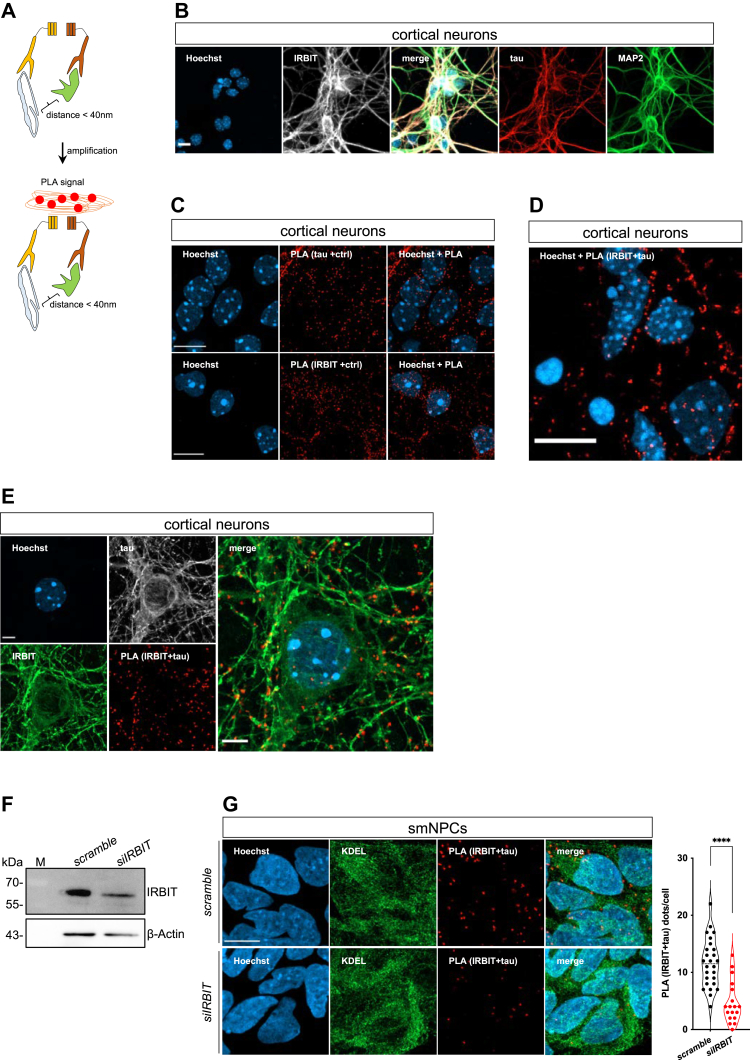
**AHCYL1/IRBIT and tau are in close proximity in cultured cells.***A*, schematic representation of the PLA method. *B*, confocal images of 7-day-old mouse primary cortical neurons stained with Hoechst-33342 (*blue*), IRBIT (*white*), tau (*red*), and MAP2 (*green*). The scale bar represents 5 μm. *C*, confocal images of primary cortical neurons stained with Hoechst-33342 (*blue*) and PLA probe (*red*). Primary antibodies were against IRBIT and tau, with +crtl indicating the presence of species-specific secondary antibodies. The scale bar represents 10 μm. *D*, representative PLA (IRBIRT + tau) staining in cortical neurons. Hoechst-33342 was used to stain the nucleus. The scale bar represents 10 μm. *E*, confocal images of mouse-derived cortical neurons stained with Hoechst-33342 (*blue*), tau (*white*), IRBIT (*green*), and PLA (IRBIT + tau; *red*). The scale bar represents 5 μm. *F*, representative immunoblots of smNPCs transfected with scramble and *siIRBIT*. Antibodies against IRBIT and β-actin (as loading control) were used. M is protein molecular marker. *G*, representative confocal images of siRNA-transfected smNPCs, which were stained with PLA for IRBIT + tau. Antibody against KDEL was used as counterstaining of the ER. Hoechst-33342 was used to image nuclei. The scale bar represents 10 μm. Quantification of PLA *dots* is reported on the *right* (Mann–Whitney test, *∗∗∗∗p* < 0.0001). AHCYL1/IRBIT, SAH hydrolase–like protein 1/IP3R-binding protein; ER, endoplasmic reticulum; KDEL, Lys–Asp–Glu–Leu; MAP2, microtubule-associated protein 2; PLA, proximity ligation assay; smNPC, small-molecule neural progenitor cell.

To detect potential interactions between endogenous proteins, we employed proximity ligation assays (PLAs), which enable *in situ* detection of proteins at close distance (<40 nm) by combining conventional immunostainings with cycles of DNA amplifications (47). To do so, cells were first incubated with two primary antibodies, followed by a pair of oligonucleotide-attached secondary antibodies. After ligation and amplification, fluorescent dot-like structures were imaged and quantified using high-resolution confocal microscopy (Fig. 2*A*). We employed mouse-derived primary cortical neurons (Fig. 2*B*) and set up our PLA-positive conditions by incubating each primary antibody (*i.e.*, IRBIT and tau) with species-specific oligonucleotide-conjugated secondary antibodies (+crtl; Fig. 2*C*). Conversely, negative controls with no fluorescent signal were obtained when primary antibodies of one species were incubated with secondary antibodies against different immunoglobulin Gs (IgGs) (data not shown). When PLA was performed by incubating both antitau and anti-IRBIT antibodies in combination with their corresponding secondary antibodies, we could detect fluorescent PLA staining as discrete puncta within the cell (Fig. 2*D*). In cortical neurons, we detected tau colocalization with MAP2, indicating that tau was also in the somatodendritic compartment because of the immature stage of the cells (Fig. 2*B*). Because of this localization pattern, PLA (IRBIT + tau) fluorescent puncta were observed in the soma as well as in neuronal projections (Fig. 2*E*). To further strengthen our evidence, we employed human smNPCs in which *AHCYL1/IRBIT* was downregulated by using synthetic siRNA oligonucleotides (Fig. 2*F*). We found that decreased expression of AHCYL1/IRBIT significantly reduced PLA signals of tau and IRBIT (Fig. 2*G*). These data indicate that tau and AHCYL1/IRBIT are in close proximity and may possibly interact in immature neurons as well as in smNPCs.

### Tau alters AHCYL1/IRBIT proximity to IP3R at the endoplasmic reticulum

**Figure 3:**
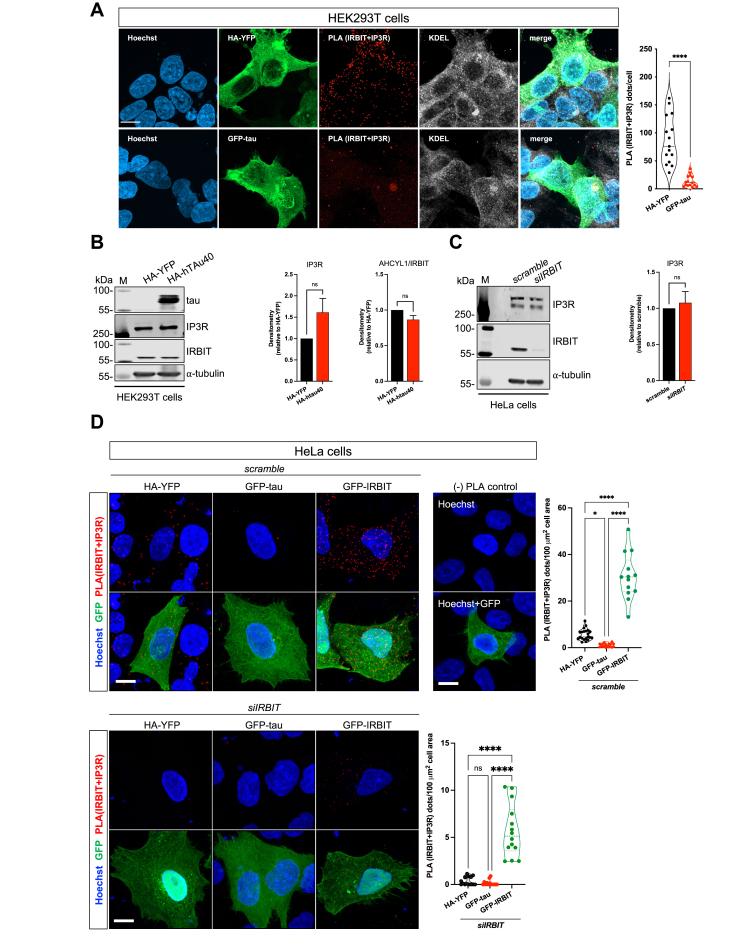
**Tau interferes with AHCYL1/IRBIT localization at the IP3R.***A*, PLA staining in HEK293T cells O/E HA-YFP or GFP-tau using primary antibodies against IRBIT and IP3R. Quantification of PLA *dots* is shown on the *right*. Data were obtained from two independent experiments and n = 15 cells/condition (unpaired *t* test, *∗∗∗∗p* < 0.0001). The scale bar represents 10 μm. *B*, representative immunoblots of HEK293T cells overexpressing HA-htau40. M is protein molecular marker. Densitometries for IP3R and AHCYL1/IRBIT expression are on the *right* (n of experiments = 4; unpaired *t* test, ns). *C*, representative immunoblots of IP3R and AHCYL1/IRBIT in HeLa cells transfected with scramble and *siIRBIT*. M is protein molecular marker. Densitometry of IP3R expression is on the *right* (n of experiments = 4; unpaired *t* test, ns). *D*, representative confocal images of HA-YFP, GFP-tau, and GFP-IRBIT overexpressing HeLa cells, which were cotransfected with scramble and *siIRBIT*. Cells were stained with Hoechst-33342 (nuclei, *blue*), anti-GFP (*green*), and PLA (IRBIT + IP3R, *red*). Quantification of PLA *dots* is shown on the *right* (n of experiments = 3; ordinary one-way ANOVA, ∗*p* < 0.05, ∗∗∗∗*p* < 0.0001, ns). Representative confocal image of a negative PLA staining is included. The scale bar represents 10 μm. AHCYL1/IRBIT, SAH hydrolase–like protein 1/IP3R-binding protein; HA, hemagglutinin; HEK293T, human embryonic kidney 293T cell line; IP3R, inositol 1,4,5-trisphosphate receptor; ns, nonsignificant; O/E, overexpression; PLA, proximity ligation assay.

We sought to explore the biological impact of tau binding to AHCYL1/IRBIT. Under physiological conditions, AHCYL1/IRBIT can bind IP3R and negatively regulate IP3R activity, thereby influencing endoplasmic reticulum (ER) Ca^2+^ release upon stimulation (42, 43, 44). We hypothesized that tau expression may influence AHCYL1/IRBIT localization to IP3R at the ER. Thus, we set up PLA experiments using a pair of validated antibodies against AHCYL1/IRBIT and IP3R. After the validation of our primary and secondary antibodies (Fig. S1, *A* and *B*), we overexpressed HA-YFP or htau40-GFP in HEK293T cells and assessed PLA (IRBIT + IP3R) signals. We found that GFP-tau (4R0N isoform) overexpressing cells had fewer PLA dots compared with controls (Fig. 3*A*), suggesting that tau overexpression (O/E) compromises AHCYL1/IRBIT localization, and possibly binding, to IP3R. Immunoblot analyses showed that neither IP3R nor AHCYL1/IRBIT expression was altered in htau40 overexpressing HEK293T cells (Fig. 3*B*). Similarly, *AHCYL1/IRBIT* downregulation did not alter IP3R level in HeLa cells (Fig. 3*C*), further ruling out that aberrant expression and/or localization of AHCYL1/IRBIT can influence IP3R expression. Next, we performed PLA (IRBIT + IP3R) in HeLa cells overexpressing GFP-tau or GFP-IRBIT, in which we transfected scramble or siRNA against *AHCYL1/IRBIT*. Compared with HA-YFP overexpressing cells (as control), GFP-tau O/E reduced the number of PLA (IRBIT + IP3R) dots in scramble-transfected cells, whereas it had no additional influence in *siIRBIT*-transfected cells (Fig. 3*D*). As expected, GFP-IRBIT O/E enhanced PLA (IRBIT + IP3R) dots and rescued the siRNA-mediated downregulation of endogenous IRBIT (Fig. 3*D*). Together, these data strongly suggest that tau can interfere with IRBIT proximity at IP3R.

**Figure 4:**
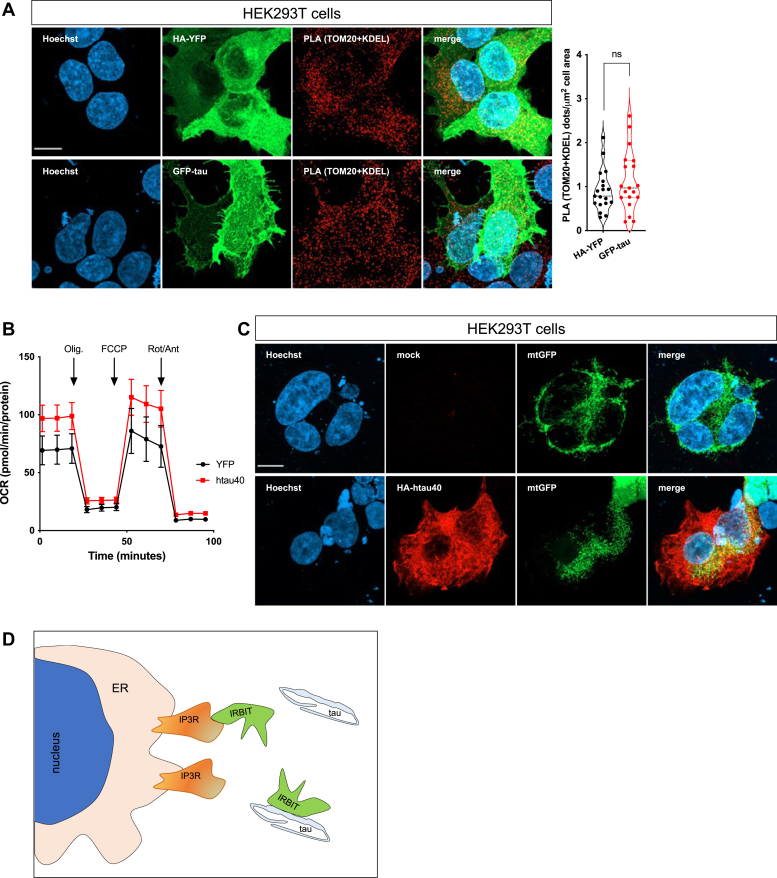
**Tau overexpression alters neither mitochondria–ER contact sites nor mitochondrial respiration.***A*, representative confocal images of HA-YFP and GFP-tau overexpressing HEK293T cells stained with PLA (TOM20 + KDEL). Quantification of PLA *dots* is shown on the *right*. Data were obtained from two independent experiments and n = 19 cells/condition (unpaired *t* test, ns). The scale bar represents 10 μm. *B* and *C*, seahorse analysis and representative confocal images of HEK293T overexpressing mitochondrial-targeted GFP and GFP-tau. The scale bar represents 10 μm. *D*, schematic representation of the interaction between tau, IRBIT, and IP3R. As a result of tau binding, IRBIT dissociates from IP3R at the ER. ER, endoplasmic reticulum; HA, hemagglutinin; HEK293T, human embryonic kidney 293T cell line; IP3R, inositol 1,4,5-trisphosphate receptor; KDEL, Lys–Asp–Glu–Leu; ns, not significant; PLA, proximity ligation assay; TOM20, translocase of outer mitochondrial membrane 20.

It was previously reported that tau O/E has negligible consequences on bulk cytosolic and mitochondrial Ca^2+^ signaling, although it moderately altered ER Ca^2+^ concentration in HeLa cells (48). With this knowledge and since AHCYL1/IRBIT as well as tau O/E can influence ER–mitochondria contacts (42, 48), we performed PLA experiments using a pair of antibodies against the outer mitochondrial membrane protein TOM20 (translocase of outer mitochondrial membrane 20) and KDEL (Lys–Asp–Glu–Leu)-containing ER proteins. When we overexpressed HA-YFP or GFP-tau in HEK293T cells and quantified the number of PLA dots, we detected comparable PLA signals in tau-overexpressing and control cells (Fig. 4*A*). Conventional seahorse experiments and confocal imaging analysis in control and tau-overexpressing HEK293T cells did not show obvious defects in mitochondrial respiration and network morphology, respectively (Fig. 4, *B* and *C*). These data suggest that tau O/E alters AHCYL1/IRBIT localization to IP3R (Fig. 4*D*), without inducing obvious changes in ER–mitochondria contacts and mitochondrial respiration.

### Tau binding to AHCYL1/IRBIT may influence autophagy

**Figure 5:**
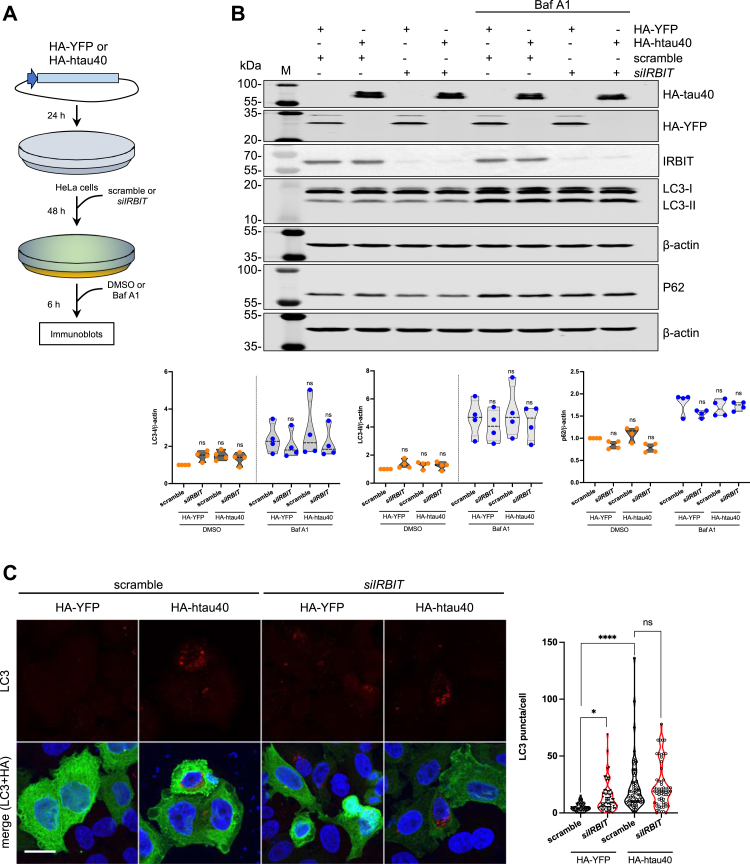
**Tau overexpression may influence autophagy in HeLa cells.***A*, schematic representation of the experimental setup. HeLa cells were transfected with HA-YFP or HA-htau40, followed by retransfection with scramble or *siIRBIT*. After 48 h, cells were incubated with DMSO or 100 nM bafilomycin A1 for 6 h. *B*, representative immunoblot for autophagy-related proteins LC3-I, LC3-II, and p62 in transfected HeLa cells. M is protein molecular marker. Quantification of LC3-I, LC3-II, and p62 protein levels normalized to β-actin. Densitometry is on the bottom. *C*, representative confocal images of HeLa cells overexpressing HA-YFP or HA-htau40 (*green*) stained for LC3-positive puncta (*red*). Nuclei (*blue*) were stained with Hoechst-33342. The scale bar represents 20 μm. The graph shows quantification of LC3-positive puncta per cell. Data in *B* and *C* were statistically analyzed *via* one-way ANOVA followed by Dunnett’s multiple comparisons test (∗∗∗∗*p* < 0.0001, ∗*p* < 0.05, ns). DMSO, dimethyl sulfoxide; HA, hemagglutinin; ns, not significant.

It was recently described that AHCYL1/IRBIT inhibits autophagy by sensing the intracellular concentration of the amino acid derivative SAH (49). We hypothesized that tau O/E may interfere with AHCYL1/IRBIT activity and, as a consequence, may alter autophagy. To explore this possibility, we transfected HeLa cells with HA-YFP (as a control) or HA-htau40, in combination with scramble or siRNA against *AHCYL1/IRBIT* (Fig. 5*A*). We ran immunoblot analyses using antibodies against the autophagic marker LC3 (50) and the autophagosome cargo SQSTM1/p62 (51). When we overexpressed hatu40 or downregulated *AHCYL1/IRBIT*, we observed minor changes in the autophagic flux, including in cells treated with the autophagic inhibitor bafilomycin A1 (Fig. 5*B*). Since transfection of plasmids did not reach an efficiency higher of 60%, we refined our study and carried out single-cell imaging analyses of LC3 puncta in HeLa cells. We found that both *AHCYL1/IRBIT* downregulation and htau40 O/E increased LC3 puncta in HeLa cells (Fig. 5*C*). Interestingly, transfection of siRNA against *AHCYL1/IRBIT* did not further increase LC3 puncta in htau40-overexpressing HeLa cells, potentially indicating that AHCYL1/IRBIT loss and tau O/E may act in the same manner on LC3 recruitment to the nascent autophagosomes. Together, these data suggest that tau may influence AHCYL1/IRBIT role in autophagy in cultured cells.

## Discussion

We herein report an unbiased protein microarray analysis that unveils potential novel tau-interacting partners. Our study describes more than 100 polypeptides that can presumably bind full-length tau *in vitro*, further expanding the list of putative tau interactors. Among these proteins, we found that recombinant htau40 interacts with a handful of previously described proteins, including DNAJ (heat shock protein 40 kD), S100β, and bridging integrator 1 (37, 38, 39, 40). Given the high affinity of the intrinsically disordered tau for many biomolecules (52), it is nevertheless surprising that we could detect only a relatively small number of polypeptides interacting with tau *in vitro* in our microarray chips. One explanation may be that recombinant proteins spotted on the chip might carry post-translational modifications of insect Sf9 cells, which may influence *in vitro*–binding features of the candidate polypeptides. Alternatively, glutathione-*S*-transferase–tagged proteins may be less mobile and assume unconventional conformations that alter physical interactions. Although we did not detect several of the well-established cytoskeletal proteins that are known to bind tau, we could find some SH3-containing proteins (*e.g.*, SAMSN1, SH3D19, UBASH3B) and kinases (YES1, LIMK1, MAPK8IP2, MKNK2, RPS6KA6, and NME1). Thus, despite the small number of tau interactors, our *in vitro* analysis may represent a reference for future studies and therefore help to develop hypothesis-driven screen of tau-interacting factors with a possible role in human pathophysiology.

We focused our attention on AHCYL1/IRBIT and carried out co-IP analyses in hippocampus lysates, transiently transfected HEK293T cells, and iPSC-derived smNPCs, thereby demonstrating that exogenous as well as endogenous tau can physically interact with AHCYL1/IRBIT. To further support our biochemical evidence, we established a PLA assay and showed that somatodendritic tau is in close proximity with AHCYL1/IRBIT in immature cortical neurons. Our evidence implies that tau can interact with AHCYL1/IRBIT in cell-free assays as well as in cultured cells. Although we did not define the interacting domains of the two molecules, we speculate that they may be within the region highly conserved between AHCYL1/IRBIT and SAH hydrolase (AHCY), given that recent data indicate that tau can also interact with AHCY in iPSC-derived glutamatergic neurons (41).

In an attempt to understand the biological implication of this interaction, we reviewed some of the initial studies describing AHCYL1/IRBIT. As mentioned earlier, AHCYL1/IRBIT is a 530-amino acid protein containing a unique N-terminal domain that is essential for binding with IP3R (43, 44). With this knowledge, we sought to test whether tau O/E could influence AHCYL1/IRBIT localization at the ER. Using another validated PLA, we report that tau can decrease the proximity of AHCYL1/IRBIT to the IP3R, implying that tau may influence the inhibitory function of AHCYL1/IRBIT exerted on IP3R. Based on these data, it would be reasonable to expect changes in calcium signaling because of the modulatory activity of AHCYL1/IRBIT on IP3R. However, prior studies had already reported that O/E of full-length tau has a minor impact on intracellular Ca^2+^ homeostasis, including ER Ca^2+^ release, in tumorigenic cells (48). Because of that, we explored other biological processes associated with IP3R activity. Since IP3R can influence mitochondria and mitochondrial homeostasis (53, 54), we assessed mitochondria–ER proximity and mitochondrial respiration in tau-overexpressing HEK293T cells; however, we could not detect any obvious difference. These data imply that tau O/E might exert an influence on AHCYL1/IRBIT function in discrete and localized subcellular regions, with a biological outcome that could not be detected through our measurements.

As a final attempt to better understand the biological meaning of our evidence, we investigated tau-AHCYL1/IRBIT interaction in the context of autophagy, since AHCYL1/IRBIT inhibits the autophagic flux as recently described (49). Autophagy is an evolutionarily conserved biological mechanism regulating bulk degradation of intracellular biomolecules and organelles. As a multistep process, autophagy encompasses a complex machinery tightly regulated at different levels by nutrient sensors that determine the biogenesis of the double membrane phagosomes, the sequestration of targeted materials, and the flux of sequestered materials to autolysosomes (55, 56). Considerable evidence is available to describe the importance of autophagy in the degradation of insoluble tau in models of AD and other tauopathies (57, 58, 59, 60, 61). While the contribution of autophagy in the homeostatic regulation of tau is evident (55, 60, 62, 63, 64, 65), it remains poorly investigated whether tau mislocalization may influence the molecular machinery that controls the autophagic flux. Thus, we set out a series of experiments in HeLa cells in which tau and AHCYL1/IRBIT expression was genetically manipulated. We report that AHCYL1/IRBIT downregulation has a mild effect on LC3-containing autophagosome accumulation that is comparable to what was observed upon tau O/E. According to our first line of experimental evidence, it seems that tau O/E does not have a considerable impact on autophagy and AHCYL1/IRBIT-dependent autophagic flux in tumorigenic cells grown in nutrient-containing media.

In summary, our study describes a new group of polypeptides that physically interact with tau *in vitro*. Among these newly identified factors, we confirm that tau binds AHCYL1/IRBIT and influences its ER localization in proximity to the IP3R. Future studies will help to better understand the implication of our observations in human pathophysiology.

## Experimental procedures

### Antibodies, PLA probes, and dyes

The following antibodies were used in this study: rabbit anti-tau, K9JA (Dako; catalog no.: A0024); mouse anti-phosphorylated tau, clone AT8 (Thermo Fisher Scientific; catalog no.: MN1020); mouse anti-tau, clone Tau46 (Sigma; catalog no.: T9450); rabbit anti-HA (Sigma; catalog no.: H6908); mouse anti-HA (Sigma; catalog no.: H9658); rabbit anti-IRBIT (Cell Signaling Technology; catalog no.: 94248); rabbit IgG (Merck Millipore; catalog no.: 12-370); mouse IgG (Merck Millipore; catalog no.: 12-371); mouse anti-α-tubulin (Sigma; catalog no.: T8203); mouse anti-β actin (Sigma; catalog no.: A5316); chicken anti-NeuN (Millipore, chicken; catalog no.: ABN91); anti-MAP2 (Abcam; catalog no.: ab5392); rabbit anti-LC3 (Sigma; catalog no.: L7543); guinea pig anti-p62 (Progen; catalog no.: GP62-C); mouse anti-IP3R-I (Santa Cruz Biotech; catalog no.: sc271197); mouse anti-β-actin (Merck; catalog no.: MAB1501); goat anti-rabbit and anti-mouse horseradish peroxidase–conjugated secondary antibodies (Promega); IRDye 800CW and 680LT secondary antibodies (LI-COR Biosciences); goat anti-mouse and goat anti-guinea pig Alexa Fluor 488, goat anti-rabbit Alexa Fluor 568, goat anti-mouse, goat anti-guinea pig and goat anti-chicken Alexa Fluor 633, and goat anti-rabbit Alexa Fluor 647 (Thermo Fisher Scientific). The following PLA probes were used for this work: Duolink *In Situ* PLA Probe Anti-Mouse Minus (Sigma; catalog no.: DUO9004); Duolink *In Situ* PLA Probe Anti-Mouse Plus (Sigma; catalog no.: DUO92001); Duolink *In Situ* PLA Probe Anti-Rabbit Minus (Sigma; catalog no.: DUO92005); Duolink *In Situ* PLA Probe Anti-Rabbit Plus (Sigma; catalog no.: DUO92002); and Duolink *In Situ* Detection Reagents Red (Sigma; catalog no.: DUO92008). Nuclei were stained with Hoechst-33342 (Thermo Fisher Scientific; catalog no.: 62249).

### Cell culture

HEK293T cells were grown in Dulbecco's modified Eagle's medium (DMEM) supplemented with 10% fetal bovine serum and 1% penicillin/streptomycin (Pen/Strep). Cells were transfected with Lipofectamine 3000 Transfection Reagent (Thermo Fisher Scientific; catalog no.: L3000015) as per the manufacturer’s instructions and were harvested 48 h after transfection. Primary cortical neurons were prepared from E14.5 embryos and seeded in DMEM supplemented with horse serum in plates previously coated with 1 mg/ml poly-l-lysine (Sigma–Aldrich; catalog no.: P1399). All culture media were replaced after 4 h with neurobasal medium supplemented with 2% B27 (Gibco; catalog no.: 17504001), 1% GlutaMAX (Gibco; catalog no.: 35050061), and 1% Pen/Strep. Cells were used 7 days after plating.

HeLa cells were transfected with HA-YFP or HA-tau40 using TurboFectin 8.0 (Origene; catalog no.: TF81001) according to the supplied protocol. After 24 h, cells were retransfected with scramble (Ambion; catalog no.: AM4635) or *siIRBIT* (Thermo Fisher Scientific; catalog no.: 4427038; ID: s651) using Lipofectamine RNAimax (Thermo Fisher Scientific; catalog no.: 13778075) and incubated for 48 h to achieve significant knockdown. Then, cells were exposed to dimethyl sulfoxide or 100 nM bafilomycin A1 (Sigma; catalog no.: 19-148) for 6 h, collected in radioimmunoprecipitation assay (RIPA) buffer (Abcam; catalog no.: ab206996) with protease inhibitor (Roche; catalog no.: 11836153001) and phosphatase inhibitor (Roche; catalog no.: 04906837001) and processed for Western blot analyses.

### Co-IP

Co-IPs were performed using Immunoprecipitation kit (Abcam; catalog no.: ab206996) following the manufacturer’s instructions. Cells were washed once with PBS and incubated with 2 mM dithiobis(succinimidyl propionate) (Thermo Fisher Scientific; catalog no.: PG82081) for 30 min for reversible crosslinking. The crosslinking reaction was stopped by incubating the cells with 25 mM Tris for 15 min. Cells were then washed with PBS and lysed with 500 μl cold nondenaturing lysis buffer (Abcam; immunoprecipitation kit; abID: ab206996). Cells we harvested were transferred to microcentrifuge tubes and incubated at 4 °C for 30 min on a rotatory mixer. After protein quantification, 500 μg of proteins were incubated with a primary antibody overnight at 4 °C on a rotatory mixer. Then, 25 μl of protein A/G Sepharose beads were added to the protein–antibody mix and incubated for 2 h at 4 °C. Beads were then collected and washed by slow speed centrifugations. Bound proteins were eluted in 40 μl 2× SDS-PAGE loading buffer (125 mM Tris–HCl [pH 6.8], 4% SDS, 20% glycerol, 10% 2-mercaptoethanol, and 0.005% bromophenol blue) and loaded on SDS-PAGE. For IP from mouse brain, hippocampus tissue was lysed with ice-cold nondenaturing lysis buffer, supplemented with protease inhibitor, and co-IP was performed as discussed previously.

### Human protein–protein interaction profiling

Protein–protein interaction profiling service was performed on ProtoArray Human Protein Microarrays, version 5.1 (Thermo Fisher Scientific). Briefly, *E. coli*-derived recombinant human full-length tau protein (htau40) was incubated at two concentrations (5 and 50 ng/μl) onto microarrays containing ∼9000 human proteins extracted from transfected insect cells. Each array contained N-terminal glutathione-*S*-transferase–tagged proteins, which were purified under nondenaturing conditions and spotted on 1 × 3 square inch glass slides coated with a thin layer of nitrocellulose. All four microarrays were saturated with blocking buffer (50 mM Hepes, 200 mM NaCl, 0.08% Triton X-100, 25% glycerol, 20 mM glutathione, 1.0 mM DTT, and 1× synthetic block) at 4 °C for 1 h under gentle shaking. Two microarrays (herein referred as protein probe microarrays) were incubated with recombinant htau40 protein diluted in probe buffer (1× PBS, 0.1% Tween-20, and 1× synthetic block), one microarray (positive control) with 50 ng/ml of array control protein (*i.e.*, yeast calmodulin kinase 1 with a biotin and V5 tags at the N terminus), and one microarray (negative control) with only probe buffer for 90 min at 4 °C. After that, microarrays were washed five times for 5 min in probe buffer at room temperature. Protein probe microarrays and negative control were incubated with rabbit anti-human tau antibody (K9JA; Dako; catalog no.: A0024, 1:20,000 dilution) in probe buffer for 90 min at 4 °C, washed five times in probe buffer, and then exposed to Alexa Fluor 647-conjugated goat anti-rabbit IgG (Thermo Fisher Scientific; catalog no.: A21244; lot no.: 1654324, 1 μg/ml in probe buffer). Positive control was exposed to anti-V5 Alexa Fluor 647–conjugated antibody for 90 min at 4 °C. All four microarrays were then washed with probe buffer, quickly rinsed in distilled water to remove residual salts, spun at 1000 rpm for 1 min to dry, and scanned using an Axon 4000B fluorescent microarray scanner (Molecular Devices). Tau-interacting proteins were considered positive candidates based on the following four criteria: (a) the fluorescent intensity value was at least 20-fold higher than the corresponding negative control; (b) the normalized fluorescent signal was greater than three standard deviations; (c) the signal-to-noise ratio was higher than 0.5; and (d) the replicate spot coefficient of variation was lower than 50%.

### Imaging analysis, sample preparation, and quantification

Immunofluorescence-stained cells and PLA experiments were imaged using an Airyscan Zeiss LSM900 confocal microscope, with a 63× oil immersion objective. Images were then deconvoluted in ZEN blue edition software (Carl Zeiss), and orthogonal projections of acquired z-stacks were used for later analysis in Fiji/ImageJ (https://fiji.sc).

For single-cell quantification of LC3 puncta, transfected HeLa cells were fixed with 4% paraformaldehyde for 20 min. Then, cells were permeabilized with methanol at −20 °C for 5 min and incubated with 2% bovine serum albumin in PBS. Cells were then incubated with primary antibody against LC3 (1:100 dilution; Sigma; catalog no.: L7543) and HA (1:100 dilution; Sigma; catalog no.: H9658) overnight at 4 °C. Cells were mounted with mounting medium (Dako).

### Immunofluorescence and PLA

For immunofluorescence, fixed cells were first blocked in a solution containing 5% normal goat serum and 0.1% Triton X-100. Following overnight incubation with primary antibodies at 4 °C, cells were washed and incubated with the specific Alexa Fluor–conjugated secondary antibodies for 2 h at room temperature and subsequently counterstained with Hoechst-33342 for 30 min. Cells were then mounted on glass slides with fluorescence mounting medium (Dako).

Fluorescence PLA was performed as described by the manufacturer, with slight modifications as previously described (66). Briefly, after antigen retrieval, cells were double permeabilized first with 0.1% Triton-X in 1× PBS for 30 min at room temperature and then with 3% H_2_O_2_ in H_2_O for 30 min at room temperature. Sections were then incubated with blocking solution (Sigma) for 1 h at 37 °C and afterward incubated with primary antibodies overnight at 4 °C. Next, specimens were incubated with secondary probes attached to oligonucleotides 1 h at 37 °C. After ligation and amplification steps, tissues were incubated with primary antibodies for cell markers overnight at 4 °C. Sections were then incubated with the corresponding Alexa Fluor–conjugated secondary antibodies and Hoechst-33342 and then mounted in glass slides for immediate image acquisition and analysis.

### Oxygen consumption rate measurements

For oxygen consumption rate (OCR) measurements, HEK293T cells were reverse transfected with HA-YFP or HA-htau40 and seeded onto cell culture microplates (Agilent Seahorse XF24) in culture media 48 h before the assay. On the day of the experiment, growth media were replaced with Seahorse XF base medium (Agilent), supplemented with 1 mM pyruvate, 10 mM glucose, and 2 mM glutamine. Cells were then equilibrated for 60 min in a CO_2_-free incubator at 37 °C. Thereafter, cells were placed into a Seahorse XFe24 Analyzer (Agilent), and, after three baseline measurements, oligomycin, carbonyl cyanide-*p*-trifluoromethoxyphenylhydrazone, and rotenone/antimycin A were successively added to profile mitochondrial respiration. The following final concentrations of compounds were used: 1 μM oligomycin, 2 μM carbonyl cyanide-*p*-trifluoromethoxyphenylhydrazone, and 0.5 μM rotenone/antimycin. At the end of OCR assessments, cells were collected, lysed, and sonicated in RIPA buffer. Protein concentrations were then determined *via* Bradford assay (Sigma), and raw OCR values were normalized to the respective protein content.

### Plasmids

The plasmid *pRP[Exp]-EGFP/Puro-CAG>hAHCYL1[NM_006621.6]* was designed and purchased from VectorBuilder. pRK5-EGFP-Tau was purchased from Addgene. All the other plasmids were available in our laboratory (PubMed identifier: 24065130). All the other plasmids were available in our laboratory (67).

### smNPC culture and differentiation

Generation of smNPCs from iPSCs was done according to a previously published protocol (68). smNPCs were cultured in Matrigel-coated 6-well plates in medium composed of DMEM-F12/neurobasal (50:50), 1% N2 supplement, 2% B27 without vitamin A (all Gibco), 0.5 μM purmorphamine, 3 μM CHIR99021 (both from Miltenyi Biotec), and 64 μg/ml LAAP (Sigma). siRNA transfection of smNPCs was performed as described for HeLa cells. To induce smNPC differentiation, medium was changed to differentiation medium containing DMEM-F12/neurobasal, 1% N2 supplement, 2% B27, 2 mM Glutamax, 1% Pen/Strep, and 1 μg/ml laminin (Sigma). Cells were differentiated under these conditions for 25 to 30 days before being collected for co-IP experiments.

### SDS-PAGE and Western blotting

Cells were harvested in PBS, lysed in RIPA buffer (Abcam; catalog no.: ab206996), and sonicated for 10 s. Samples were immediately centrifuged at 10,000*g* at 4 °C for 20 min, and supernatants were used for protein quantification using Bradford Reagent (Sigma). Lysates were treated with 4× loading buffer (250 mM Tris–HCl [pH 6.8], 8% SDS, 40% glycerol, 20% 2-mercaptoethanol, and 0.01% bromophenol blue) and boiled at 95 °C for 5 min before loading on SDS-PAGE. Separated proteins on polyacrylamide gels were transferred onto nitrocellulose membranes and incubated in blocking buffer (5% bovine serum albumin) or 5% dried skim milk; Tris-buffered saline (TBS)–Tween (50 mM Tris–HCl, 150 mM NaCl, and 0.1% Tween-20) for 1 h at room temperature. Then, nitrocellulose membranes were incubated with primary antibodies in blocking buffer overnight at 4 °C. The next day, membranes were washed three times in TBS–Tween, followed by 1 h incubation at room temperature with anti-rabbit (LI-COR Biosciences; IRDye 800CW, 1:10,000 dilution) or antimouse (LI-COR Biosciences; IRDye 680RD; 1:10,000 dilution) secondary antibodies. Membranes were washed three times in TBS–Tween and one time in PBS. Immunoblots were visualized using an Odyssey Infrared Imaging System (LI-COR Biosciences). Densitometry was performed using the image processing software Fiji.

### Statistical analysis

GraphPad Prism software (GraphPad Software, Inc) was used for statistical analyses. Specific comparison between experimental groups was analyzed using Student’s *t* test. For three or more groups, one-way ANOVA (Dunnett’s post hoc correction) was employed. Data were expressed as means ± SEM. The number of biological replicates is indicated in the legends to the figures.

## Data availability

All data are contained within the article.

## Supporting information

This article contains supporting information (Fig. S1 and Table S1).

## Conflict of interest

The authors declare that they have no conflicts of interest with the contents of this article.

## Figures and Tables

**Supplemental Figure S1:**